# Efficient Precoding and Power Allocation Techniques for Maximizing Spectral Efficiency in Beamspace MIMO-NOMA Systems

**DOI:** 10.3390/s23187996

**Published:** 2023-09-20

**Authors:** Yongfei Liu, Lu Si, Yuhuan Wang, Bo Zhang, Weizhang Xu

**Affiliations:** 1 Engineering Research Center of Digital Audio & Video Ministry of Education, Communication University of China, Beijing 100024, China; 2 State Key Laboratory of Media Convergence & Communication, Communication University of China, Beijing 100024, China; 3 Datang Mobile Communications Equipment Co., Ltd., Beijing 100083, China

**Keywords:** beamspace MIMO-NOMA, sum rate, precoding, power allocation, FP

## Abstract

Beamspace MIMO-NOMA is an effective way to improve spectral efficiency. This paper focuses on a downlink non-orthogonal multiple access (NOMA) transmission scheme for a beamspace multiple-input multiple-output (MIMO) system. To increase the sum rate, we jointly optimize precoding and power allocation, which presents a non-convex problem. To solve this difficulty, we employ an alternating algorithm to optimize the precoding and power allocation. Regarding the precoding subproblem, we demonstrate that the original optimization problem can be transformed into an unconstrained optimization problem. Drawing inspiration from fraction programming (FP), we reconstruct the problem and derive a closed-form expression of the optimization variable. In addition, we effectively reduce the complexity of precoding by utilizing Neumann series expansion (NSE). For the power allocation subproblem, we adopt a dynamic power allocation scheme that considers both the intra-beam power optimization and the inter-beam power optimization. Simulation results show that the energy efficiency of the proposed beamspace MIMO-NOMA is significantly better than other conventional schemes.

## 1. Introduction

With the coverage of mobile connections expanded, wireless communications systems are facing an escalating demand for data traffic, which poses challenges in terms of spectral efficiency and energy efficiency. Non-orthogonal multiple access (NOMA) technology has emerged as a key solution for improving spectral efficiency and supporting massive links, as it enables multiple users to share the same spectrum resource simultaneously. The application of NOMA in conventional terrestrial communication systems, benefiting from its superior spectral efficiency capability and capacity to accommodate massive connectivity, has been thoroughly investigated in many aspects [1]. Additionally, beamspace multiple-input multiple-output (beamspace MIMO) as another key technology also has several advantages. It leverages the abundant spectrum resources in the millimeter wave band, enabling terminal equipment to achieve high-rate data transmission. Furthermore, by employing large-scale MIMO, beamspace MIMO forms directional beams with high gain, effectively mitigating the challenge of substantial signal transmission path loss inherent in millimeter wave communication. Consequently, beamspace MIMO is recognized as a promising technology for future wireless communications [2]. Does this mean we can combine the NOMA and beamspace MIMO technologies to effectively leverage their advantages in the power and spatial domains, leading to improved spectral efficiency? The answer is affirmative. Specifically, considering the characteristics of these two technologies, beamspace MIMO requires a large number of radio frequency (RF) chains, which leads to high energy consumption and renders the all-digital structure unsuitable for direct application [3]. Moreover, in beamspace MIMO, the number of supported users cannot exceed the number of RF chains, thereby limiting the system’s capacity to accommodate users. However, NOMA excels in increasing the number of system access users. Consequently, the integration of NOMA with mmWave massive MIMO, known as beamspace MIMO-NOMA, has emerged as a promising solution for significantly increasing the number of connections and further enhancing spectral efficiency. This approach has garnered growing research interest [4].

### 1.1. Prior Works

Typically, the optimization of precoding and power allocation designs is considered a means to improve the performance of beamspace MIMO-NOMA systems. These problems have been investigated jointly or partially. However, the presence of inter-beam and intra-beam interference makes these problems non-convex and challenging to solve [5]. Fortunately, researchers have developed efficient algorithms to tackle these challenges.

Some works have focused on separately designing precoding or power allocation to improve performance. In [6], the authors propose a ZF precoding scheme to mitigate interference between users and employ the Karush–Kuhn–Tucker (KKT) conditions to investigate the power allocation problem for maximizing the sum rate. Furthermore, [7] explores energy efficiency maximization through power allocation and presents a two-layer iterative algorithm to tackle the non-convex optimization problem. The outer layer converts the original fractional objective function by using the Dinkelbach method, while the inner layer utilizes alternating optimization to solve the transformed problem. In [8], the authors introduce a low-complexity iterative algorithm called mean square error-based dynamic power allocation algorithm (MSE-DPA), which achieves near-perfect performance. Ref. [9] proposes a criterion based on correlation for user pairing to reduce inter-user interference, with ZF precoding applied to the paired users. The results demonstrate that the proposed scheme achieves higher spectral efficiency compared to the conventional scheme. In [10], the main objective is to design a low-complexity hybrid precoder (HP), where the authors propose a symmetric successive over-relaxation (SSOR) algorithm combined with complex regularized zero-forcing (CRZF) linear precoding.

In addition, a significant focus of the work is to design a joint optimization scheme for precoding and power allocation to enhance system performance. In [11], the authors adopt a ZF-based precoding scheme to mitigate inter-beam interference and propose a dynamic power allocation method based on minimum mean square error (MMSE) to maximize the achievable sum rate in beamspace MIMO-NOMA systems. Ref. [12] addresses the limitations of complicated successive interference cancellation (SIC) that were disregarded in [11]. Based on the ZF beamforming technique, the power allocation optimization problem is represented as a fractional programming (FP) problem, which was transformed into a convex optimization problem using sequential convex approximation (SCA) and second-order cone (SOC) transformation. In [13], the authors formulate a joint hybrid beamforming and power allocation problem to maximize the sum rate. They employ the approximate ZF method to design the digital beamforming for minimizing inter-group interference and solve the analog beamforming problem with a constant-modulus constraint using a proposed boundary-compressed particle swarm optimization algorithm. In [14], the authors design ZF precoding matrices and evaluate power allocation coefficients based on optimal spectral efficiency to mitigate intra-beam interference. Additionally, they derive a tight closed-form formula for optimal spectral efficiency using KKT analysis. In [15], from the perspective of spectral efficiency, the authors propose a joint optimization framework and employ the quadratic transformation (QT) method to convert the non-convex power allocation problem into a convex problem. They also design an iterative approach to obtain optimal power allocation and digital beamforming. In [16], the authors propose a hybrid precoder that combines user channel alignment and the ZF algorithm to enhance the SINR. Furthermore, they address the non-convex optimization problem by transforming it into a convex optimization problem for inter-cluster power allocation, which can be solved by using the KKT conditions.

### 1.2. Motivations and Contributions

While the aforementioned research contributions have established a strong foundation for beamspace MIMO-NOMA, further investigation and improvements are still necessary to address practical considerations. Firstly, there is scope for enhancing the optimization of key performance indicators that impact spectral efficiency through various methodologies. Secondly, there is a need for research to focus on reducing computational complexity while improving spectral efficiency simultaneously, which remains an open area of exploration. These observations have inspired our primary research objectives in this study. In this work, our main goal is to maximize the sum rate of beamspace MIMO-NOMA in downlink communications and propose an optimal design scheme for joint precoding and power allocation, building upon the previous research. Against this backdrop, we emphasize the following four aspects that constitute the contributions of our paper:

- Firstly, we employ block optimization to optimize the joint problem of precoding and power allocation in beamspace MIMO-NOMA systems. In the precoding optimization part, we demonstrated that the original constrained problem can be transformed into an unconstrained problem. Moreover, we elucidated the quantitative relationship between the solutions of the original problem and the equivalent unconstrained problem. For the power allocation part, we adopted a dynamic power allocation method based on a joint power optimization problem, taking into account power optimization within and between beams.
- Secondly, we devised a precoding scheme based on FP to decouple the optimization variables, effectively transforming the unconstrained problem into three equivalent subproblems. Subsequently, we derived closed expressions for the optimization variables.
- Thirdly, as the number of antennas at the BS and the number of users accessing the system increase, the hardware and signal processing complexity also escalates. Since the precoding optimization algorithm involves complex matrix inversion operations, its calculation complexity is 𝒪$\left(N_{RF}{}^{3}\right)$, which grows cubically with the increase in the number of RF connections. To mitigate this complexity, we utilized the Neumann series expansion (NSE) method to approximate the inverse of the precise matrix and expand the lower-order terms, thereby reducing the complexity of the matrix inversion operation to 𝒪$\left(N_{RF}{}^{2}\right)$.
- Finally, we validated the performance of the proposed scheme through simulation. The results demonstrated that the algorithm significantly improves spectral efficiency. Furthermore, the simulation results confirmed that the proposed precoding and power allocation scheme outperforms the benchmark methods.

### 1.3. Organization and Notations

The remainder of the paper is organized as follows. Section 2 outlines the system model of beamspace MIMO-NOMA. Based on this model, Section 3 formulates the maximum sum rate problem, and an introduction to the proposed algorithm is provided. Section 4 presents the simulation results to evaluate the performance. Finally, Section 5 concludes the paper.

Notation: Denote ℂ as the set of complex numbers, and $Re\left\{\cdot \right\}$ as the real part. We use the superscript to denote the Hermitian transpose of a matrix and overline the complex conjugate. The bold lower-case letter denotes a vector; the bold upper-case letter denotes a matrix; the calligraphic upper-case letter denotes a set. $I_{n}$ denotes the identity matrix of dimension n.${\left(\cdot \right)}^{T},{\left(\cdot \right)}^{H},{\left(\cdot \right)}^{-1}$ and ${\left\|\cdot \right\|}_{F}$ denote transpose, Hermitian transpose, inversion, and Frobenius norm operations, respectively.

## 2. System Model and Problem Formulation

In this section, we first review the beamspace MIMO system model, followed by a detailed description of the beamspace MIMO-NOMA system model.

### 2.1. System Model of Beamspace MIMO

**Figure 1:**
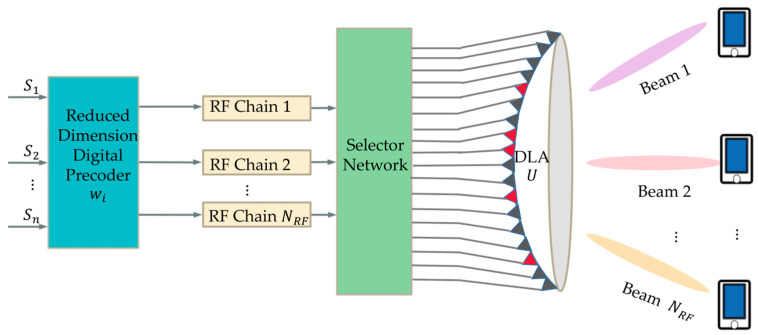
The system model of beamspace MIMO architecture.

As illustrated in Figure 1, the system depicted represents a single-cell downlink mmWave MIMO communication system. The BS is equipped with N antennas and $N_{RF}$ RF chains, serving K randomly distributed single-antenna users simultaneously [17]. Employing the usual uniform linear array (ULA) structure, utilizing a well-designed lens antenna array at the BS. The received signal vector $y={\left[y_{1},y_{2},\cdots ,y_{K}\right]}^{T}$ is represented as:

(1) $$y=H^{H}WPs+n,$$

where $s={\left[s_{1},s_{2},\cdots ,s_{K}\right]}^{T}\in C^{K\times 1}$ represents the transmitted signal vector for all K users satisfied with $E\left({ss}^{H}\right)=I_{K}$, $P=diag\left(p_{1},p_{2},\cdots ,p_{K}\right)$ is the diagonal power allocation matrix, $W=\left[w_{1},w_{2},\cdots ,w_{K}\right]\in C^{N\times K}$ is the precoding matrix, and $H=\left[h_{1},h_{2},\cdots ,h_{K}\right]\in C^{N\times K}$ is the Rayleigh fading channel matrix, where $h_{k}\in C^{N\times 1}$ denotes the channel vector between the BS and the kth user. In addition, n is the noise vector that follows the distribution $CN\left(0,\sigma^{2}I_{K}\right)$. We consider the widely used Saleh–Valenzuela channel model for mmWave communications, $h_{k}$ can be represented as

(2) $$h_{k}=\beta_{k,0}a\left(\theta_{k,0}\right)+\overset{L}{\underset{l=1}{\sum }}\beta_{k,l}a\left(\theta_{k,l}\right),$$

where $\beta_{k,0}$ denotes the LoS complex path gain, while $a\left(\theta_{k,0}\right)$ represents the array steering vector for the LoS path, similarly, $\beta_{k,l}$ and $a\left(\theta_{k,l}\right)$ denote the complex gain and steering vector for the lth NLoS path, respectively. Furthermore, L denotes the number of NLoS paths.

For the typical ULA, the expression of $a\left(\varphi \right)$ is can be expressed as follows [18]:

(3) $$a\left(\theta \right)=\frac{1}{\sqrt{N}}{\left[e^{-j2\pi \theta m}\right]}_{m\in J(N)},$$

where $J(N)=\left\{i-(N-1)/2,i=0,1,\cdots ,N-1\right\}$ is a symmetric set of indices centered around zero. The spatial direction of the channel is defined as $\theta =\frac{d}{\lambda }\sin\left(\varphi \right)$, λ represents the wavelength, $d=\frac{\lambda }{2}$ denotes the antenna spacing, and φ denotes the physical direction of the corresponding path satisfying $-\frac{\pi }{2}\leq \theta \leq \frac{\pi }{2}$.The lens antenna array serves as a discrete Fourier transformation matrix U, defined as

(4) $$U={\left[a\left({\bar{\theta }}_{1}\right),a\left({\bar{\theta }}_{2}\right),\cdots ,a\left({\bar{\theta }}_{N}\right)\right]}^{H},$$

where ${\bar{\theta }}_{n}=\frac{1}{N}\left(n-\frac{N+1}{2}\right)$ for n=1,2,⋯,N are the predefined spatial directions.

Then, the received signal vector $\bar{y}$ in the beamspace MIMO systems is given by

(5) $$\bar{y}=H^{H}U^{H}WPs+n={\bar{H}}^{H}WPs+n,$$

where $\bar{H}=UH$ is the beamspace channel matrix. We employ the classic maximum-magnitude-based beam selection method to choose a subset of the N orthogonal beams to serve all K users without obvious performance loss [19]. Consequently, the number of RF chains is reduced from N to $N_{RF}$. Thus, the received signal can be written as

(6) $$\bar{y}={\bar{H}}_{r}^{H}W_{r}Ps+n,$$

where ${\bar{H}}_{r}=\bar{H}\left(m,:\right)$, m∈M is the dimension-reduced beamspace channel matrix with size $\left|M\right|\times K$, and M is the index set of selected beams. It is important to note that in this system, one RF chain generates one beam, resulting in the number of selected beams $\left|M\right|$ being equal to the number of RF chains $N_{RF}$ [11]. In addition, the dimension-reduced digital precoding matrix $W_{r}$, has a size of $\left|M\right|\times K$. Since $W_{r}$ has a smaller row dimension compared to the original precoding matrix W, the number of required RF chains can be significantly reduced [20].

Notwithstanding, reducing the number of RF chains also presents a challenge of limited connections. To overcome this fundamental limit, a novel transmission scheme known as beamspace MIMO-NOMA, which combines the concept of NOMA with beamspace MIMO, has been proposed. By incorporating NOMA into beamspace MIMO systems, both spectral efficiency and connection density can be further enhanced [6].

### 2.2. System Model of Beamspace MIMO-NOMA

**Figure 2:**
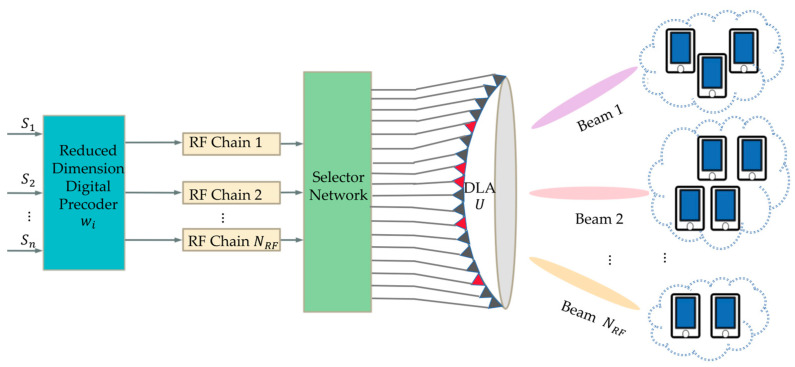
The system model of beamspace MIMO-NOMA architecture.

As shown in Figure 2, this is a typical beamspace MIMO-NOMA wireless communication system. We consider that there are $N_{RF}$ groups assigned to provide service, and we denote the set of users $S_{m}$ served by the mth beam with $S_{m}\cap S_{n}=\emptyset$ for m≠n and $\sum_{m=1}^{N_{RF}}\left|S_{m}\right|=K$. The received signal ${\hat{y}}_{m,n}$ of the nth user in the mth beam can be expressed as follows:

(7) $${\hat{y}}_{m,n}=\underset{desired\;signal}{\underset{︸}{h_{m,n}^{H}w_{m}\sqrt{p_{m,n}}s_{m,n}}}+\underset{intra-beam\;interferences}{\underset{︸}{\left(\overset{n-1}{\underset{k=1}{\sum }}h_{m,n}^{H}w_{m}\sqrt{p_{m,k}}s_{m,k}+\overset{\left|S_{m}\right|}{\underset{k=n+1}{\sum }}h_{m,n}^{H}w_{m}\sqrt{p_{m,k}}s_{m,k}\right)}}+\underset{\mathrm{int}er-beam\;interferences}{\underset{︸}{\overset{N_{RF}}{\underset{l\neq m}{\sum }}\overset{\left|S_{l}\right|}{\underset{k=1}{\sum }}h_{m,n}^{H}w_{l}\sqrt{p_{l,k}}s_{l,k}}}+\underset{noise}{\underset{︸}{v_{m,n}}},$$

where $s_{m,n}$ is the transmitted signal for the nth user in the mth beam with normalized power, and $p_{m,n}$ is the corresponding transmitted power, $w_{m}=W_{r}\left(:,m\right)$ represents the mth beam digital precoding vector, and $v_{m,n}\sim CN\left(0,\sigma^{2}\right)$ refers to the noise. Based on the principle of NOMA, intra-beam interference can be mitigated by utilizing SIC. Supposing that ${\left\|h_{m,1}\right\|}^{2}\geq {\left\|h_{m,2}\right\|}^{2}\geq \cdots \geq {\left\|h_{m,\left|S_{m}\right|}\right\|}^{2}$ for $m=1,2,\cdots ,N_{RF},$ within the same beam, the ith user can sequentially detect the jth user (for all j>i) and remove the detected signals from its received signals [21]. In the mth beam, after employing SIC to decode the nth user’s signal, the remaining received signal can be expressed as follows:

(8) $$y_{m,n}=\underset{desired\;signal}{\underset{︸}{h_{m,n}^{H}w_{m}\sqrt{p_{m,n}}s_{m,n}}}+\underset{intra-beam\;interferences}{\underset{︸}{\overset{n-1}{\underset{k=1}{\sum }}h_{m,n}^{H}w_{m}\sqrt{p_{m,k}}s_{m,k}}}+\underset{\mathrm{int}er-beam\;interferences}{\underset{︸}{\overset{N_{RF}}{\underset{l\neq m}{\sum }}\overset{\left|S_{l}\right|}{\underset{k=1}{\sum }}h_{m,n}^{H}w_{l}\sqrt{p_{l,k}}s_{l,k}}}+\underset{noise}{\underset{︸}{v_{m,n}}}.$$

Therefore, the signal-to-interference-plus-noise ratio (SINR) at the nth user in the mth beam can be expressed as follows:

(9) $$\gamma_{m,n}=\frac{{\left\|h_{m,n}^{H}w_{m}\right\|}_{2}^{2}p_{m,n}}{\xi_{m,n}},$$

where

(10) $$\xi_{m,n}=\overset{n-1}{\underset{k=1}{\sum }}{\left\|h_{m,n}^{H}w_{m}\right\|}_{2}^{2}p_{m,k}+\overset{N_{RF}}{\underset{l\neq m}{\sum }}\overset{\left|S_{l}\right|}{\underset{k=1}{\sum }}{\left\|h_{m,n}^{H}w_{l}\right\|}_{2}^{2}p_{l,k}+\sigma^{2}.$$

Hence, the corresponding achievable rate can be expressed as follows:

(11) $$R_{m,n}=\log_{2}\left(1+\gamma_{m,n}\right).$$

Consequently, the overall achievable sum rate of the beamspace MIMO-NOMA scheme is:

(12) $$R_{sum}=\overset{N_{RF}}{\underset{m=1}{\sum }}\overset{\left|S_{m}\right|}{\underset{n=1}{\sum }}R_{m,n},$$

Indeed, precoding optimization helps mitigate inter-beam interference, but intra-beam interference endures within beamspace MIMO-NOMA systems. Power allocation effectively mitigates this inter-beam interference, thus enhancing overall system performance. It is noteworthy that expressions (9)–(11) illustrate the substantial influence of power allocation parameters $\left\{p_{m,n}\right\}$ and precoding vectors $\left\{w_{m}\right\}$ on maximizing the sum rate. Thus, the system performance can be further enhanced through the meticulous design of precoding and power allocation strategies. Jointly optimizing precoding and power allocation is pivotal for maximizing overall system performance. While this may add complexity, thoughtful design, and analysis allow for performance improvements without imposing significantly higher computational demands. In the following section, we will explore these ideas in greater detail.

## 3. Alternating Optimization of Beam-Specific Digital Precoding and Power Allocation

In this section, we begin by formulating the optimization problem. Next, we present an alternating optimization method to obtain the solution for beam-specific digital precoding. Finally, we maximize the achievable sum rate by solving the joint power optimization problem using a dynamic power allocation scheme.

### 3.1. Problem Formulation

Our objective is to maximize the achievable sum rate problem by jointly optimizing the beam-specific digital precoding and power allocation, while adhering to the maximum transmit power constraint of the BS. The optimization problem can be formulated as follows:

(13) $$\begin{array}{c} P_{1}:\underset{\left\{p_{m,n}\right\}\left\{w_{m}\right\}}{\max}R_{sum}\\ \;s.t.C_{1}:p_{m,n}\geq 0,\forall m,n,\\ C_{2}:\overset{N_{RF}}{\underset{m=1}{\sum }}\overset{\left|S_{m}\right|}{\underset{n=1}{\sum }}p_{m,n}{\left\|w_{m}\right\|}^{2}\leq P_{max}. \end{array}$$

Obviously, three problems need to be addressed to optimize $P_{1}$. As shown in (9), the presence of both intra-beam interference and inter-beam interference in the system results in the optimization variable $\left\{p_{m,n}\right\}$ and $\;\left\{w_{m}\right\}$ appears in both the nomination and denominator of $\;\gamma_{m,n}$. Consequently, the problem becomes a non-convex optimization problem that is difficult to solve directly. Furthermore, it is highly nonlinear. Additionally, the optimization of precoding $\left\{w_{m}\right\}$ is performed at the beam level, while the optimization of power allocation $\left\{p_{m,n}\right\}$ is carried out at the user level. This implies that both aspects are difficult to optimize simultaneously.

To tackle the complexity of the original problem $P_{1}$, we decompose it into two sub-problems: ${\tilde{P}}_{beam}$ and $P_{power}$ for optimization. For the sub-problem ${\tilde{P}}_{beam}$, we first convert the constrained optimization problem into an unconstrained optimization problem. Then, we employ the FP algorithm to handle the NP-hard problem, leading to the derivation of a closed expression for precoding W. Additionally, we leverage the NSE to reduce the complexity of the precoding process. As for the sub-problem of power allocation, we utilize a dynamic power allocation scheme to obtain a closed-form expression for the power distribution, ensuring lower complexity.

### 3.2. The Proposed Beam-Specific Digital Precoding Optimization

In this subsection, we focus on optimizing the beam-specific digital precoding vectors $\left\{w_{m}\right\}$ for a given set of power allocation parameters $\left\{p_{m,n}\right\}$. To accomplish this, we transform the non-convex precoding optimization problem into an unconstrained optimization problem. To be specific, the precoding problem can be formulated as follows:

(14) $$\begin{array}{c} {\tilde{P}}_{beam}:\underset{\left\{w_{m}\right\}}{\max}R_{sum}\\ s.t.C_{1}:\sum_{m=1}^{N_{RF}}\sum_{n=1}^{\left|S_{m}\right|}p_{m,n}{\left\|w_{m}\right\|}^{2}\leq P_{\max}. \end{array}$$

Specifically, inspired by [22], we establish the following definition and proposition.

**Definition 1.**  *(Trivial Stationary Point): If a point X satisfying HX = 0, which results in a zero-sum rate, we say that it is a trivial stationary point of the original problem*$P_{1}$.

**Proposition 1.**  *Any nontrivial stationary point of problem *${\tilde{P}}_{beam}$ *must satisfy the constraint *$C_{1}$ *with equality.*

**Proof.**  See Appendix A. □

According to Proposition 1, it can be inferred that:

(15) $$\frac{\sum_{x=1}^{N_{RF}}\sum_{y=1}^{\left|S_{x}\right|}p_{x,y}{\left\|w_{x}\right\|}^{2}}{P_{\max}}=1.$$

Hence, the problem ${\tilde{P}}_{beam}$ can be transformed into the following unconstrained form:

(16) $${\bar{P}}_{beam}:\underset{\left\{w_{m}\right\}}{\max}\sum_{m=1}^{N_{RF}}\sum_{n=1}^{\left|S_{m}\right|}\log_{2}\left(1+\frac{{\left\|h_{m,n}^{H}w_{m}\right\|}_{2}^{2}p_{m,n}}{\sum_{k=1}^{n-1}{\left\|h_{m,n}^{H}w_{m}\right\|}_{2}^{2}p_{m,k}+\sum_{l\neq m}^{N_{RF}}\sum_{k=1}^{\left|S_{l}\right|}{\left\|h_{m,n}^{H}w_{l}\right\|}_{2}^{2}p_{l,k}+\frac{\sum_{x=1}^{N_{RF}}\sum_{y=1}^{\left|S_{x}\right|}p_{x,y}{\left\|w_{x}\right\|}^{2}}{P_{\max}}\sigma^{2}}\right).$$

We can express the objective function as follows:

(17) $$f_{beam}\left(w\right)=\sum_{m=1}^{N_{RF}}\sum_{n=1}^{\left|S_{m}\right|}\log_{2}\left(1+\frac{{\left\|h_{m,n}^{H}w_{m}\right\|}_{2}^{2}p_{m,n}}{{\tilde{\xi }}_{m,n}}\right),$$

where

(18) $${\tilde{\xi }}_{m,n}=\sum_{k=1}^{n-1}{\left\|h_{m,n}^{H}w_{m}\right\|}_{2}^{2}p_{m,k}+\sum_{l\neq m}^{N_{RF}}\sum_{k=1}^{\left|S_{l}\right|}{\left\|h_{m,n}^{H}w_{l}\right\|}_{2}^{2}p_{l,k}+\frac{\sum_{x=1}^{N_{RF}}\sum_{y=1}^{\left|S_{x}\right|}p_{x,y}{\left\|w_{x}\right\|}^{2}}{P_{\max}}\sigma^{2}.$$

The following proposition establishes the relationship between ${\tilde{P}}_{beam}$ and ${\bar{P}}_{beam}$.

**Proposition 2.**  *The following relationship exists between the optimal solution* ${\tilde{w}}^{o}$ *of the problem* ${\tilde{P}}_{beam}$ *and the optimal solution* ${\bar{w}}^{o}$ *of the new unconstrained optimization problem* ${\bar{P}}_{beam}$.

(19) $$\;{\tilde{w}}_{m}^{o}=\sqrt{\frac{P_{max}}{\sum_{m=1}^{N_{RF}}\sum_{n=1}^{\left|S_{m}\right|}p_{m,n}{\left\|{\bar{w}}_{m}^{o}\right\|}^{2}}}{\bar{w}}_{m}^{o}.$$

**Proof.**  See Appendix B.□

This implies that if we find solution ${\bar{w}}^{o}$, then solution ${\tilde{w}}^{o}$ can be obtained according to Proposition 2.

Obviously, the objective function $f_{beam}\left(w\right)$ remains non-convex, making it difficult to solve in polynomial time. To address this, we employ the Lagrangian dual transform to reframe the unconstrained problem ${\bar{P}}_{beam}$, as demonstrated below [23].

(20) $$\begin{array}{c} {\bar{P}}_{beam}:\underset{\left\{w_{m}\right\}\left\{u_{m,n}\right\}}{\min}f_{beam}^{LD}\left(w,u\right),\\ s.t.C_{1}:u_{m,n}\in \mathbb{R},\forall m, \end{array}$$

where u refers to a set of auxiliary variables $\left\{u_{m,n}\right\}$, and the objective function of problem ${\bar{P}}_{beam}$ is formulated as follows:

(21) $$f_{beam}^{LD}\left(w,u\right)=\sum_{m=1}^{N_{RF}}\sum_{n=1}^{\left|S_{m}\right|}-\left(\log_{2}(1+u_{m,n})-u_{m,n}+\frac{\left(1+u_{m,n}\right){\left\|h_{m,n}^{H}w_{m}\right\|}_{2}^{2}p_{m,n}}{{\left\|h_{m,n}^{H}w_{m}\right\|}_{2}^{2}p_{m,n}+{\tilde{\xi }}_{m,n}}\right).$$

When $w_{m}$ is held fixed, the optimal $u_{m,n}$ can be obtained by solving $\frac{\partial f_{beam}^{LD}\left(w,u\right)}{\partial u_{m,n}}=0$, i.e.,

(22) $$u_{m,n}^{o}=\frac{{\left\|h_{m,n}^{H}w_{m}\right\|}_{2}^{2}p_{m,n}}{{\tilde{\xi }}_{m,n}}.$$

Now, we incorporate $u_{m,n}^{o}$ into (21) and obtain

(23) $$f_{beam}^{LD}\left(w,u\right)=\overset{N_{RF}}{\underset{m=1}{\sum }}\overset{\left|S_{m}\right|}{\underset{n=1}{\sum }}-\left[const\left(u\right)+\frac{\left(1+u_{m,n}\right){\left\|h_{m,n}^{H}w_{m}\right\|}_{2}^{2}p_{m,n}}{{\left\|h_{m,n}^{H}w_{m}\right\|}_{2}^{2}p_{m,n}+{\tilde{\xi }}_{m,n}}\right],$$

where $const\left(u\right)=\log_{2}\left(1+u_{m,n}\right)-u_{m,n}$ is a constant term. Applying the multidimensional quadratic transform further transforms (23) and leads to the following expression:

(24) $$f_{beam}^{MQ}\left(w,u,v\right)=\overset{N_{RF}}{\underset{m=1}{\sum }}\overset{\left|S_{m}\right|}{\underset{n=1}{\sum }}\left[-const\left(u\right)-2\sqrt{\left(1+u_{m,n}\right)p_{m,n}}Re\left\{h_{m,n}^{H}w_{m}v_{m,n}^{*}\right\}+{\left|v_{m,n}\right|}^{2}\left({\left\|h_{m,n}^{H}w_{m}\right\|}_{2}^{2}p_{m,n}+{\tilde{\xi }}_{m,n}\right)\right].$$

where v is the collection $\left\{v_{m,n}\right\}$. With $u_{m,n}$ fixed, the optimal $v_{m,n}$ can also be determined by setting $\frac{\partial f_{beam}^{MQ}\left(w,u,v\right)}{\partial v_{m,n}}=0$, and the optimal value $v_{m,n}^{o}$ can be expressed as follows:

(25) $$v_{m,n}^{o}=\frac{\sqrt{\left(1+u_{m,n}\right)p_{m,n}}h_{m,n}^{H}w_{m}}{{\left\|h_{m,n}^{H}w_{m}\right\|}_{2}^{2}p_{m,n}+{\tilde{\xi }}_{m,n}}.$$

Likewise, with the other variables fixed, the optimal $w_{m}$ satisfies the expression $\frac{\partial f_{beam}^{MQ}\left(w,u,v\right)}{\partial w_{m}}=0$, i.e.,

(26) $$\begin{array}{c} w_{m}^{o}={\left(\overset{\left|S_{m}\right|}{\underset{n=1}{\sum }}{\left|v_{m,n}\right|}^{2}h_{m,n}h_{m,n}^{H}\sum_{k=1}^{n}p_{m,k}+\overset{N_{RF}}{\underset{l\neq m}{\sum }}\overset{\left|S_{l}\right|}{\underset{n=1}{\sum }}{\left\|v_{l,n}\right\|}_{2}^{2}\overset{\left|S_{m}\right|}{\underset{k=1}{\sum }}h_{l,n}h_{l,n}^{H}p_{m,k}+\overset{N_{RF}}{\underset{x=1}{\sum }}\overset{\left|S_{x}\right|}{\underset{y=1}{\sum }}{\left|v_{x,y}\right|}^{2}\frac{\sum_{i=1}^{\left|S_{m}\right|}p_{m,i}}{P_{max}}\sigma^{2}I_{N_{RF}}\right)}^{-1}\\ \cdot \overset{\left|S_{m}\right|}{\underset{n=1}{\sum }}\sqrt{\left(1+u_{m,n}\right)p_{m,n}}h_{m,n}v_{m,n}. \end{array}$$

The proposed algorithm is summarized in Algorithm 1. Unfortunately, although $N_{RF}$ is much smaller than K, the matrix inversion in the expression of $w_{m}^{o}$ still remains high-dimensional, resulting in computational complexity of 𝒪$\left(N_{RF}{}^{3}\right)$ in each iteration, which may result in significant processing delays. To address this issue, the NSE has been explored as an alternative for approximating matrix inversion [24], we leverage the NSE to simplify the matrix inversion of $w_{m}^{o}$ as follows.

Letting

(27) $$A=\overset{\left|S_{m}\right|}{\underset{n=1}{\sum }}{\left|v_{m,n}\right|}^{2}h_{m,n}h_{m,n}^{H}\sum_{k=1}^{n}p_{m,k}+\overset{N_{RF}}{\underset{l\neq m}{\sum }}\overset{\left|S_{l}\right|}{\underset{n=1}{\sum }}{\left\|v_{l,n}\right\|}_{2}^{2}\overset{\left|S_{m}\right|}{\underset{k=1}{\sum }}h_{l,n}h_{l,n}^{H}p_{m,k}+\overset{N_{RF}}{\underset{x=1}{\sum }}\overset{\left|S_{x}\right|}{\underset{y=1}{\sum }}{\left|v_{x,y}\right|}^{2}\frac{\sum_{i=1}^{\left|S_{m}\right|}p_{m,i}}{P_{max}}\sigma^{2}I_{N_{RF}},$$

we can observe that the matrix A exhibits diagonal dominance. In such cases, the inversion of A can be equivalently expressed as follows [25]:

(28) $$A^{-1}=\overset{\infty }{\underset{n=0}{\sum }}{\left(P^{-1}\left(P-A\right)\right)}^{n}P^{-1}.$$

By decomposing the matrix A as A=D+E, where D is a diagonal matrix consisting of the main diagonal elements of A, and E is a hollow matrix consisting of the remaining elements. Replace P in (28) with  D and rewrite it as follows

(29) $$A^{-1}=\overset{\infty }{\underset{n=0}{\sum }}{\left(D^{-1}\left(D-A\right)\right)}^{n}D^{-1}=\overset{\infty }{\underset{n=0}{\sum }}{\left(-D^{-1}E\right)}^{n}D^{-1}.$$

Due to the high complexity of the full NSE algorithm, the truncated NSE, which aims to retain only the first k  orders (k+1 terms) of the Neumann series, is a more commonly used approach. The specific formula can be expressed as follows:

(30) $$A^{-1}\approx A_{k}^{-1}=\overset{k}{\underset{n=0}{\sum }}{\left(-D^{-1}E\right)}^{n}D^{-1}.$$

It should be noted that as the unfolding order increases (denoted as ‘k>1’), the computational complexity of the proposed NSE-based algorithm may exceed the complexity of 𝒪$\left(N_{RF}{}^{3}\right)$. Therefore, to strike a balance between closely approximating the original precoding while reducing complexity, we choose k=1, then $A^{-1}\approx D^{-1}-D^{-1}ED^{-1}$, we have the following expression [26]

(31) $$w_{m}^{o}\approx \left(D^{-1}-D^{-1}ED^{-1}\right)\overset{\left|S_{m}\right|}{\underset{n=1}{\sum }}\sqrt{\left(1+u_{m,n}\right)p_{m,n}}h_{m,n}v_{m,n}.$$

Based on this estimation, the NSE-level approximation algorithm can reduce the computational complexity from 𝒪$\left(N_{RF}{}^{3}\right)$ to 𝒪$\left(N_{RF}{}^{2}\right)$. By combining the aforementioned updates, Algorithm 1 provides a detailed description of the proposed precoding optimization algorithm.
**Algorithm 1** Proposed Precoding Framework.**Input:** Beamspace channel vectors: $h_{m,n}$ for ∀m,n; Power allocation parameters: $p_{m,n}$ for ∀m,n; Noise variance: $\sigma^{2}$; Maximum iteration times: $T_{max}$. **Output:** Optimal precoding vectors: $w_{m}^{o}$ for ∀m; 1. t=0. 2. **while**
$t<T_{max}$ **do** 3. Obtain the optimal $\left\{u_{m,n}^{\left(t\right)}\right\}$ according to (22); 4. Obtain the optimal $\left\{v_{m,n}^{\left(t\right)}\right\}$ according to (25); 5. Obtain the optimal $\left\{w_{m}^{\left(t\right)}\right\}$ according to (31); 6. t=t+1 7. **end while** 8. **return**
$w_{m}^{o}=\sqrt{\alpha }w_{m}^{\left(t\right)}$ for ∀m.

In Algorithm 1, it can be demonstrated that the computational complexity is primarily determined by line 5. Within each iteration, the complexity of obtaining the optimal values $\left\{u_{m,n}^{\left(t\right)}\right\}$ in (22) and $\left\{v_{m,n}^{\left(t\right)}\right\}$ in (25) is linear in the number of RF chains, i.e., 𝒪$\left(N_{RF}\right)$. Additionally, the complexity of finding the optimal value $\left\{w_{m}^{\left(t\right)}\right\}$ in (31) is 𝒪$\left(N_{RF}{}^{2}\right)$, due to the utilization of NSE. Consequently, the computational complexity is significantly lower than the complexity of 𝒪$\left(N_{RF}{}^{3}\right)$ stated in (26).

### 3.3. The Adopted Optimization Power Allocation

The initial optimization problem $P_{1}$ can be transformed into the following problem when $\left\{w_{m}\right\}$ is fixed.

(32) $$\begin{array}{c} P_{power}:\underset{\left\{p_{m,n}\right\}}{\max}R_{sum}\\ s.t.C_{1}:p_{m,n}\geq 0,\forall m,n,\\ C_{2}:\overset{N_{RF}}{\underset{m=1}{\sum }}\overset{\left|S_{m}\right|}{\underset{n=1}{\sum }}p_{m,n}{\left\|w_{m}\right\|}^{2}\leq P_{max}. \end{array}$$

Note that the problem remains challenging. To address this difficulty, we introduce Lemma 1 to simplify problem $P_{power}$.

**Lemma 1.**  *Let* $f\left(a\right)=-\frac{ab}{\ln2}+\log_{2}a+\frac{1}{\ln2}$ *and* $a\in {\mathbb{R}}^{1\times 1}$ *be a positive scalar, we have* 

(33) $$-\log_{2}b=\underset{a>0}{\max}f\left(a\right),$$

 *where the optimal solution of* a *is* $a^{o}=\frac{1}{b}$.

**Proof.**  Since $f\left(a\right)$ is a convex function, and the optimal solution of $f\left(a\right)$ can be obtained by setting $\frac{\partial f\left(a\right)}{\partial a}=0$, we can derive that

(34) $$-\frac{b}{\ln2}+\frac{1}{a\ln2}=0\Rightarrow a=\frac{1}{b},$$

where the maximum value of $f\left(a\right)$ is $-\log_{2}b$. □

Moreover, if we use the minimum mean square error (MMSE) to estimate $s_{m,n}$, then have the following expression:

(35) $$e_{m,n}=E\left\{{\left|s_{m,n}-c_{m,n}y_{m,n}\right|}^{2}\right\},$$

where $c_{m,n}\in {\mathbb{C}}^{1\times 1}$ denotes the channel equalization coefficient, $y_{m,n}$ is defined previously in (8). Substituting into (35), we obtain:

(36) $$e_{m,n}={\left|1-c_{m,n}\sqrt{p_{m,n}}h_{m,n}^{H}w_{m}\right|}^{2}+{\left|c_{m,n}\right|}^{2}\xi_{m,n}.$$

According to [11], the optimal equalization coefficient $c_{m,n}$ can be obtained by the following formula:

(37) $$c_{m,n}=\arg\underset{c_{m,n}}{\min}e_{m,n},$$

and $c_{m,n}$ can be calculated by $\frac{\partial e_{m,n}}{\partial c_{m,n}}=0$, then we have

(38) $$c_{{}_{m,n}}^{o}={\left(\sqrt{p_{m,n}}h_{m,n}^{H}w_{m}\right)}^{*}{\left({\left\|h_{m,n}^{H}w_{m}\right\|}_{2}^{2}p_{m,n}+\xi_{m,n}\right)}^{-1}.$$

Substituting (38) into (36), we can obtain the optimal MMSE expression as follows:

(39) $$e_{m,n}^{o}=1-p_{m,n}{\left\|h_{m,n}^{H}w_{m}\right\|}_{2}^{2}{\left({\left\|h_{m,n}^{H}w_{m}\right\|}_{2}^{2}p_{m,n}+\xi_{m,n}\right)}^{-1}.$$

According to the extension of the Sherman-Morrison-Woodbury formula [27],

(40) $${\left(A+BCD\right)}^{-1}=A^{-1}-A^{-1}B{\left(I+{CDA}^{-1}B\right)}^{-1}{CDA}^{-1}.$$

Thus, ${\left(1+\gamma_{m,n}\right)}^{-1}$ can be reformulated as

(41) $${\left(1+\gamma_{m,n}\right)}^{-1}=1-p_{m,n}{\left\|h_{m,n}^{H}w_{m}\right\|}_{2}^{2}{\left({\left\|h_{m,n}^{H}w_{m}\right\|}_{2}^{2}p_{m,n}+\xi_{m,n}\right)}^{-1}.$$

We observe that the expression (41) has the same form as the MMSE expression (39). i.e., we have

(42) $${\left(1+\gamma_{m,n}\right)}^{-1}=\underset{c_{m,n}}{\min}e_{m,n}={\left(e_{m,n}\right)}^{o}$$

Using Lemma 1, we can equivalently rewrite $P_{power}$ as

(43) $$\begin{array}{c} {\hat{P}}_{power}:\underset{\left\{p_{m,n}\right\}}{\max}\overset{N_{RF}}{\underset{m=1}{\sum }}\overset{\left|S_{m}\right|}{\underset{n=1}{\sum }}\underset{\left\{c_{m,n}\right\}}{\max}\underset{\left\{a_{m,n}\right\}}{\max}\left(-\frac{a_{m,n}e_{m,n}}{\ln2}+\log_{2}a_{m,n}+\frac{1}{\ln2}\right)\\ s.t.C_{1}:p_{m,n}\geq 0,\forall m,n,\\ C_{2}:\overset{N_{RF}}{\underset{m=1}{\sum }}\overset{\left|S_{m}\right|}{\underset{n=1}{\sum }}p_{m,n}{\left\|w_{m}\right\|}^{2}\leq P_{max}. \end{array}$$

where $a_{m,n}>0$ is an introduced slack variable. We propose to iteratively optimize $\left\{p_{m,n}\right\}$, $\left\{c_{m,n}\right\}$ and $\left\{a_{m,n}\right\}$ by using the alternating optimization algorithm. The optimal solution can be obtained by:

(44) $$a_{m,n}^{o}=\frac{1}{e_{m,n}^{o}}.$$

After obtaining the optimal values $c_{m,n}^{o}$ and $a_{m,n}^{o}$ in the iteration, the optimal value $p_{m,n}^{o}$ can be obtained by solving the following problem:

(45) $$\begin{array}{c} {\tilde{P}}_{power}:\underset{\left\{p_{m,n}\right\}}{\max}\overset{N_{RF}}{\underset{m=1}{\sum }}\overset{\left|S_{m}\right|}{\underset{n=1}{\sum }}a_{m,n}e_{m,n}\\ s.t.C_{1}:p_{m,n}\geq 0,\forall m,n,\\ C_{2}:\overset{N_{RF}}{\underset{m=1}{\sum }}\overset{\left|S_{m}\right|}{\underset{n=1}{\sum }}p_{m,n}{\left\|w_{m}\right\|}^{2}\leq P_{max}. \end{array}$$

We observe that ${\tilde{P}}_{power}$ is a convex optimization problem, which can be solved by using the following Lagrange function:

(46) $$L\left(p,\lambda \right)=\overset{N_{RF}}{\underset{m=1}{\sum }}\overset{\left|S_{m}\right|}{\underset{n=1}{\sum }}a_{m,n}\left({\left|1-c_{m,n}\sqrt{p_{m,n}}h_{m,n}^{H}w_{m}\right|}^{2}+{\left|c_{m,n}\right|}^{2}\xi_{m,n}\right)+\lambda \left(\overset{N_{RF}}{\underset{m=1}{\sum }}\overset{\left|S_{m}\right|}{\underset{n=1}{\sum }}p_{m,n}{\left\|w_{m}\right\|}^{2}-P_{max}\right)$$

where λ≥0. Then, the Karush–Kuhn–Tucker (KKT) condition of problem ${\tilde{P}}_{power}$ can be obtained as follows.

(47) $$\frac{\partial L\left(p,\lambda \right)}{\partial p_{m,n}}=-\frac{a_{m,n}}{\sqrt{p_{m,n}}}\;Re\left\{c_{m,n}h_{m,n}^{H}w_{m}\right\}+\overset{\left|S_{m}\right|}{\underset{y=n}{\sum }}a_{m,y}{\left|c_{m,y}\right|}^{2}{\left\|h_{m,y}^{H}w_{m}\right\|}_{2}^{2}+\overset{N_{RF}}{\underset{x\neq m}{\sum }}\overset{\left|S_{x}\right|}{\underset{k=1}{\sum }}a_{x,k}{\left|c_{x,k}\right|}^{2}{\left\|h_{x,k}^{H}w_{m}\right\|}_{2}^{2}+\lambda {\left\|w_{m}\right\|}^{2}=0,$$

(48) $$\lambda \left(\overset{N_{RF}}{\underset{m=1}{\sum }}\overset{\left|S_{m}\right|}{\underset{n=1}{\sum }}p_{m,n}{\left\|w_{m}\right\|}^{2}-P_{max}\right)=0.$$

Finally, the optimal solution $p_{m,n}^{o}$ from (45) can be found as follows:

(49) $$p_{m,n}^{o}={\left(\frac{a_{m,,n}Re\left\{c_{m,n}h_{m,n}^{H}w_{m}\right\}}{\sum_{y=n}^{\left|S_{m}\right|}a_{m,y}{\left|c_{m,y}\right|}^{2}{\left\|h_{m,y}^{H}w_{m}\right\|}_{2}^{2}+\sum_{x\neq m}^{N_{RF}}\sum_{k=1}^{\left|S_{x}\right|}a_{x,k}{\left|c_{x,k}\right|}^{2}{\left\|h_{x,k}^{H}w_{m}\right\|}_{2}^{2}+\lambda {\left\|w_{m}\right\|}^{2}}\right)}^{2}.$$

We can see that the values of $\left\{c_{m,n}^{o}\right\}$, $\left\{a_{m,n}^{o}\right\}$ and $\left\{p_{m,n}^{o}\right\}$ obtained in each iteration are closed optimal solutions because (37), (33) and (45) are all convex after a sequence of transformations. The iterative update of $\left\{c_{m,n}^{o}\right\}$, $\left\{a_{m,n}^{o}\right\}$ and $\left\{p_{m,n}^{o}\right\}$ will only increase or maintain the objective function in (43). A monotonically growing sequence of objective function values in (43) can be obtained through iterative updating. However, it has an upper bound because of the transmission power restriction. Therefore, the proposed iterative optimization algorithm for power allocation will converge to a stationary solution of problem ${\hat{P}}_{power}$. The power allocation optimization technique is described in detail in Algorithm 2. We summarize the proposed algorithm in Algorithm 3.
**Algorithm 2** Proposed Power Allocation Framework.**Input:** Beamspace channel vectors: $h_{m,n}$ for ∀m,n; Precoding vectors: $w_{m}$ for ∀m; Noise variance: $\sigma^{2}$; Maximum iteration times: $T_{max}$. **Output:** Optimal power allocation vectors: $p_{m,n}^{o}$ for ∀m,n; 1. t=0. 2. **while** $t<T_{max}$ **do** 3. Obtain the optimal $\left\{c_{m,n}^{\left(t\right)}\right\}$ according to (38); 4. Obtain the optimal $\left\{a_{m,n}^{\left(t\right)}\right\}$ according to (44); 5. Obtain the optimal $\left\{p_{m,n}^{\left(t\right)}\right\}$ according to (49); 6. t=t+1 7. **end while** 8. **return** $p_{m,n}^{o}=p_{m,n}^{\left(t\right)}$ for ∀m,n.


**Algorithm 3** Proposed Joint Precoding and Power Allocation Framework.**Input:** Maximum iteration times: $T_{max}$. **Initialize:** Power allocation $p_{m,n}^{0}$ for ∀m,n **Output:** Optimal power allocation vectors and precoding vectors: $w_{m}^{o}$, $p_{m,n}^{o}$ for ∀m,n; 1. t=0. 2. **while** $t<T_{max}$ **do** 3. Find the optimal beamfoming vectors $w_{{}_{m}}^{\left(t\right)}$ given $p_{m,n}^{\left(t-1\right)}$ by Algorithm 1; 4. Find the optimal allocation vectors $p_{m,n}^{\left(t\right)}$ given $w_{{}_{m}}^{\left(t\right)}$ by Algorithm 2; 5. t=t+1 6. **end while** 7. **return** $w_{m}^{o}=\sqrt{\alpha }w_{m}^{\left(t\right)}$, $p_{m,n}^{o}=p_{m,n}^{\left(t\right)}$ for ∀m,n.


The computational complexity of the proposed algorithm mainly arises from the iteration part. We observe that in each iteration, the complexity of obtaining the optimal values $\left\{c_{m,n}^{o}\right\}$ in (38) and $\left\{a_{m,n}^{o}\right\}$ in (44) is linear with the number of users, i.e., 𝒪$\left(K\right)$. λ in (48) can be obtained by using the Newton or bisection methods, both of which have a complexity of 𝒪$\left(K^{2}\log_{2}\delta \right)$, where δ represents the desired accuracy. The overall complexity of the suggested power allocation algorithm can be calculated to be 𝒪$\left(T_{max}K^{2}\log_{2}\delta \right)$, where $T_{max}$ is the maximum number of repetitions. Therefore, the complexity of the proposed joint precoding design and power allocation optimization algorithm is 𝒪$\left(T_{max}K^{2}\log_{2}\delta +T_{max}N_{RF}{}^{3}\right)$. While the computational complexity of the algorithm without NSE processing is 𝒪$\left(T_{max}K^{2}\log_{2}\delta +T_{max}N_{RF}{}^{4}\right)$.

## 4. Simulation Result

The performance of the proposed joint optimization algorithm for the mmWave beamspace MIMO-NOMA scheme is evaluated by using numerical simulations in this section.

### 4.1. Simulation Setup

In this paper, we consider a typical single-cell downlink mmWave massive MIMO system. The BS is equipped with a ULA of N=256 transmit antennas that communicate with K users simultaneously. The system bandwidth is assumed to be 1 Hz, and the total transmit power is set to P=32mW (15 dBm) [11]. For all users’ channels, we assume L=1 LoS component and L=2 NLoS components, where $\beta_{k}^{\left(0\right)}\sim CN\left(0,1\right),\beta_{k}^{\left(l\right)}\sim CN\left(0,10^{-1}\right)$ for 1≤l≤L, $\theta_{k}^{\left(0\right)}$ and $\theta_{k}^{\left(l\right)}$ follows a uniform distribution within $\left[-\frac{1}{2},\frac{1}{2}\right]$ for 1≤l≤L. The SNR is set as $\frac{P}{\sigma^{2}}$, the maximum number of iterations $T_{max}=50$.

We consider the following four typical mmWave massive MIMO solutions for comparison, and we aim to use the same system configuration in these systems to conduct a fair comparison: “traditional fully digital MIMO” (FDM), “traditional beamspace MIMO” (BM), “traditional MIMO-OMA”(MO), in particular, we compared our approach with the reference [11], which is a particularly classic and highly effective method based on a “beamspace MIMO-NOMA” (BMN) system, as a benchmark.

We evaluated the performance in terms of energy efficiency and spectral efficiency of each of the four baseline systems mentioned above. According to [20], energy efficiency can be expressed as:

(50) $$\varepsilon_{EE}=\frac{R_{sum}}{P_{t}+N_{RF}P_{RF}+N_{RF}P_{SW}+P_{BB}}$$

where $P_{t}$ represents the total transmit power, $P_{RF}$ represents the power consumed by each RF, $P_{SW}$ represents the power consumed by each switch, and $P_{BB}$ represents the power consumed at the baseband. For the parameters, we have adopted the following common values: $R_{\mathrm{RF}}=300\mathrm{mW}$, $P_{\mathrm{SW}}=5\mathrm{mW}$ and $P_{\mathrm{BB}}=200\mathrm{mW}$.

### 4.2. Simulation Results

The performance evaluation of the proposed MIMO-NOMA system was carried out in three different cases: performance comparison at different SNRs, performance comparison at different numbers of users, and performance comparison at different numbers of antennas.

➀ Comparison of performance with different SNRs

**Figure 3:**
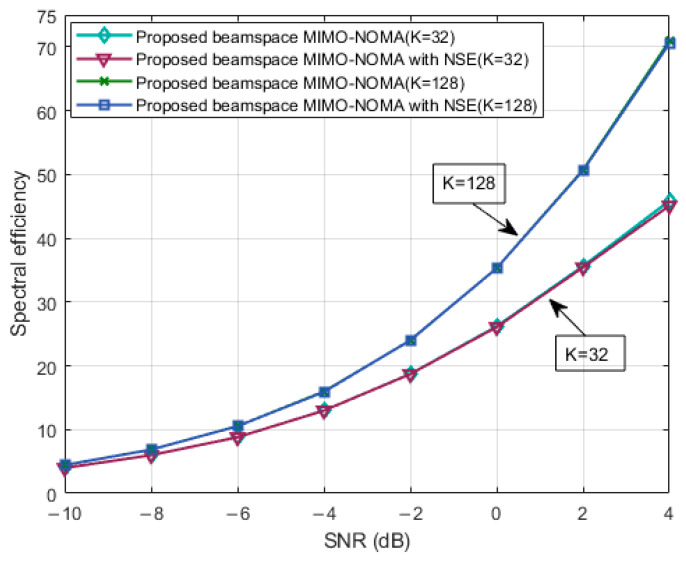
Spectrum efficiency comparison versus SNRs of the two schemes with different users.

Figure 3 depicts the comparison of spectral efficiency versus SNRs with K=32 and K=128. As the SNR increases, both sets of curves demonstrate an increase in spectral efficiency. The proposed optimization structures namely proposed ‘BMN’ and proposed ‘beamspace MIMO-NOMA with NSE’ (BMNN), exhibited very similar results in terms of spectral efficiency growth. This indicates that our precoding scheme, approximated by the NSE, not only reduced the complexity of the original algorithm but also achieved comparable performance. These findings highlight the effectiveness of the NSE approximation algorithm.

**Figure 4:**
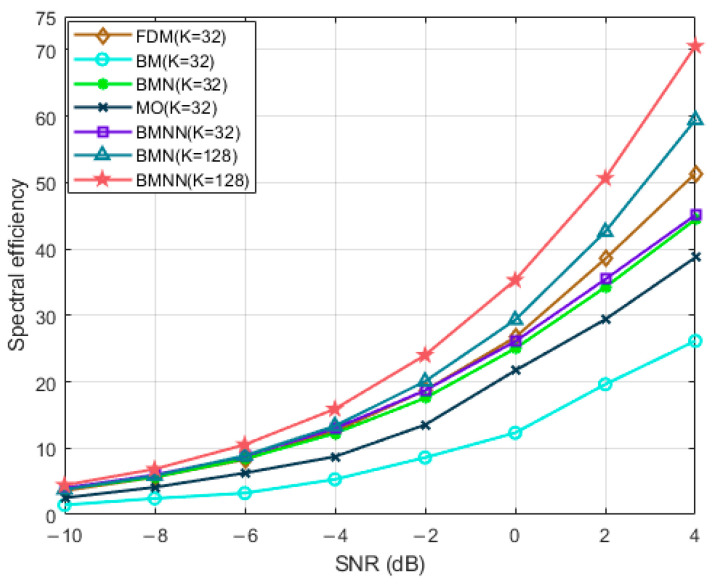
Spectrum efficiency comparison versus SNRs with different users.

Figure 4 presents a comparison of spectral efficiency versus SNRs for the proposed system and the baseline systems. In particular, we compared the spectral efficiency of the proposed algorithm in the beamspace MIMO-NOMA system with the classical BMN [11] algorithm, both for 128 users and 32 users. The results indicate that in both scenarios, BMNN outperformed BMN [11], with the advantage becoming more pronounced as the number of users increased. When there were 32 users, the proposed BMNN scheme outperformed the BMN [11], BM, and MO schemes in terms of spectral efficiency. Particularly, compared to BMN [11], the performance gain of BMNN came mainly from the optimization of precoding for different beams in the first stage. Moreover, the proposed BMNN exhibited significantly better performance than BM, benefiting from the integration of beamspace MIMO and NOMA technologies, which enabled simultaneous service to multiple users within each beam and effectively improved spectral efficiency. Since NOMA can achieve higher spectral efficiency than OMA, it is evident that the proposed BMNN also outperforms MO in terms of spectral efficiency.

**Figure 5:**
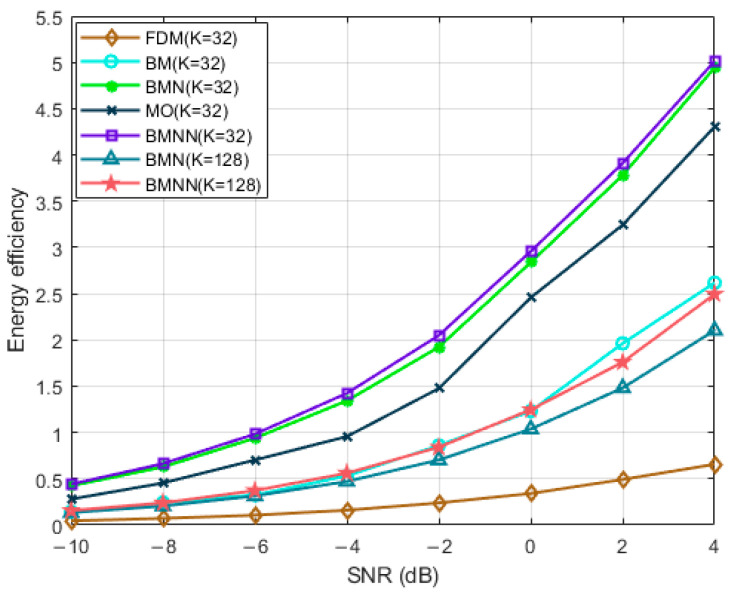
Energy efficiency comparison versus SNRs with different users.

Figure 5 illustrates the comparison of energy efficiency versus SNRs with K = 32 and K=128 users for the proposed system and the baseline systems. It can be clearly seen that increasing SNR can lead to a substantial growth in energy efficiency, and within the same system, for both 32 users and 128 users, our algorithm outperformed BMN [11]. Furthermore, in different systems with 32 users, the energy efficiency of the proposed BMNN was higher than that of the other four baseline systems. Specifically, compared to BM, our proposed BMNN achieved higher energy efficiency, by integrating NOMA and beamformed MIMO, allowing each beam to serve multiple users.

➁ Comparison of performance with different users

The aforementioned results were obtained while considering varying SNR, however, in real communication systems, especially in massive MIMO systems, the number of accessed users plays a significant role. Therefore, we further investigated the spectrum efficiency performance of the two proposed solutions under different user scenarios.

**Figure 6:**
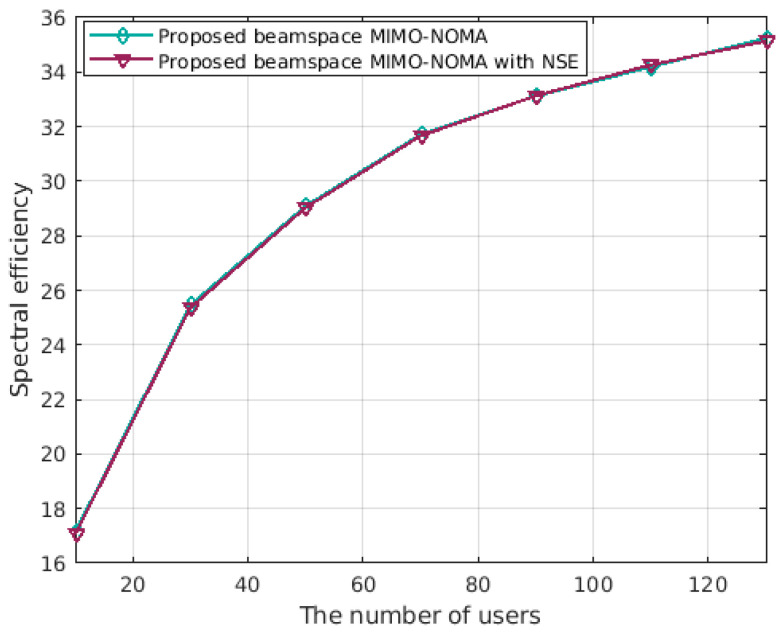
Spectrum efficiency comparison versus users of the two schemes at SNR=0 dB.

Figure 6 depicts how spectrum efficiency varies with the number of users. Both curves exhibit an upward trend with increasing user count, and the spectrum efficiency growth curves of the two proposed optimization structures yield similar results.

**Figure 7:**
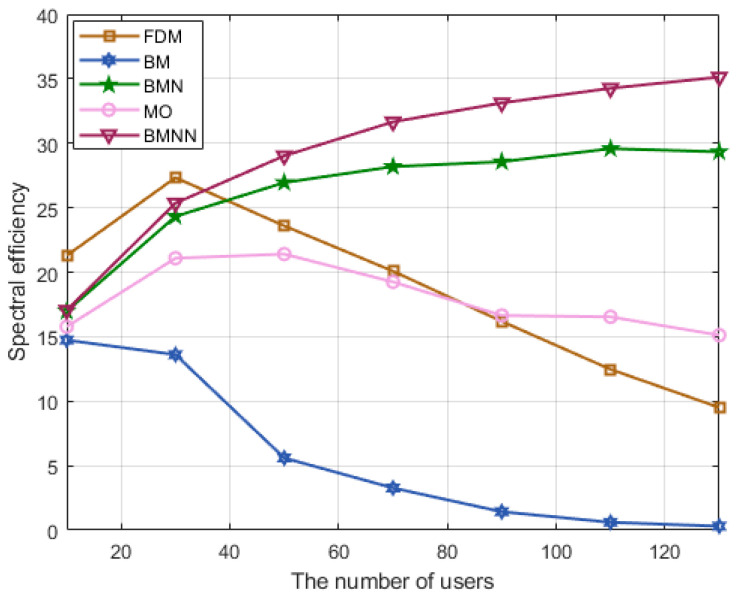
Spectrum efficiency comparison versus users at SNR=0 dB.

Figure 7 illustrates a comparison of the spectrum efficiency of the four schemes under different user scenarios at 0 dB. The BMNN scheme outperformed the BMN [11], BM, and MO schemes. Moreover, compared to the traditional BM schemes, the BMNN optimization scheme proposed in this study further improved spectrum efficiency.

**Figure 8:**
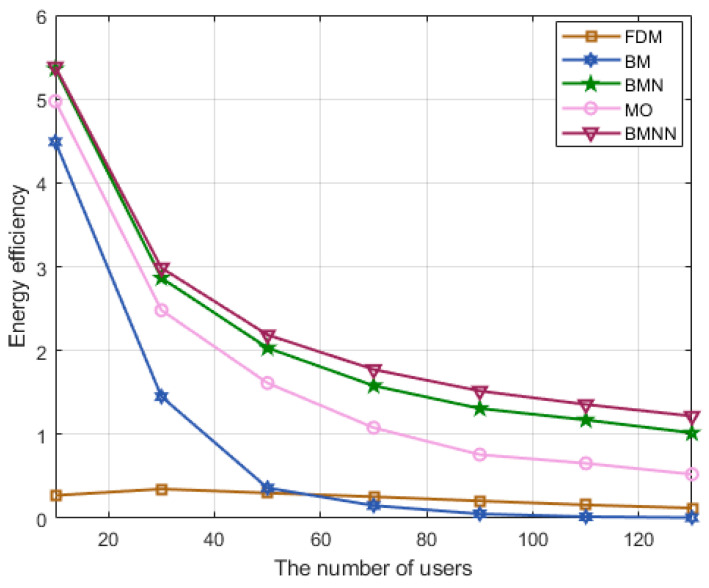
Energy efficiency comparison versus users at SNR=0 dB.

Figure 8 displays the energy efficiency performance for all considered schemes as the number of users increases. It is obvious that the proposed algorithm remained superior among the five schemes, which proves the effectiveness of the proposed scheme. Another noteworthy observation is that the performance of our proposed BMNN algorithm surpassed that of BMN [11] in terms of energy efficiency. This is mainly attributed to the fact that BMN [11] utilizes the ZF algorithm commonly employed in many studies in the precoding part, whereas our proposed algorithm optimizes the precoding parameters, thereby validating the necessity of optimizing precoding design parameters in our algorithm.

**Figure 9:**
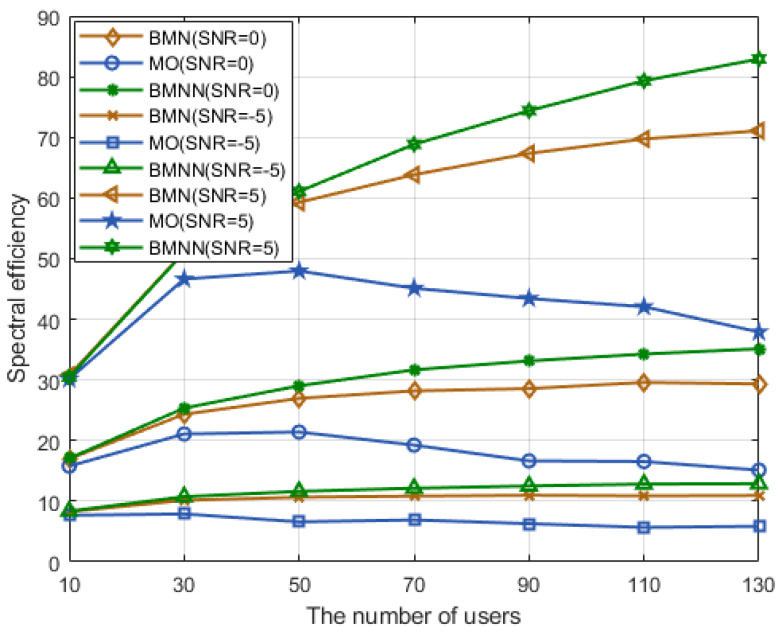
Spectral efficiency comparison versus users with different SNRs.

Figure 9 shows how spectral efficiency varies with an increasing number of users at SNR levels of −5 dB, 0 dB, and 5 dB. It is important to note that, across all these SNR conditions, the BMNN algorithm we propose consistently outperformed the other schemes, and its superiority becomes even more pronounced as the SNR increases.

➂ Comparison of performance with different antennas

**Figure 10:**
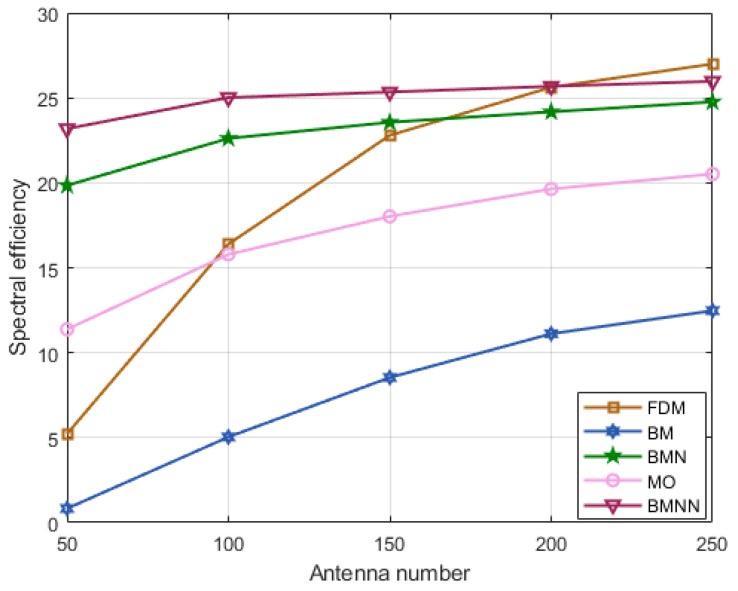
Spectral efficiency comparison versus antennas number at SNR=0 dB.

From Figure 10, it can be observed that, BMNN exhibited a clear advantage over other algorithms until the number of antennas increases to 200. Beyond this point, the spectrum efficiency of the FDM algorithm surpassed that of the others. This is primarily attributed to the increase in the number of antennas in the FDM algorithm. With more antennas, precise beamforming becomes possible, allowing for more accurate signal focusing. This allows signals to be aimed more accurately at the receivers, reducing signal scattering and interference, ultimately leading to improved spectral efficiency.

**Figure 11:**
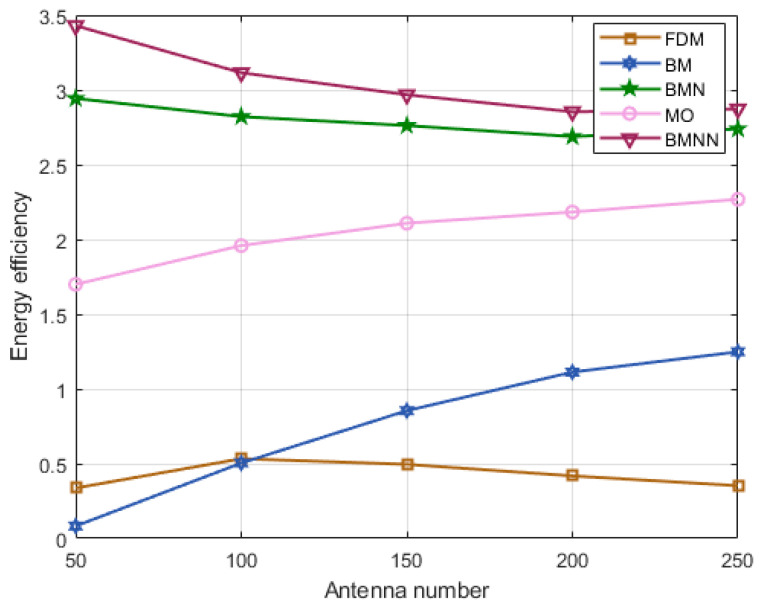
Energy efficiency comparison versus antennas number at SNR=0 dB.

However, it is worth noting that the FDM algorithm typically requires more hardware and signal processing resources, which can lead to higher power consumption. As Figure 11 corroborates, the energy efficiency of the FDM tends to be lower. Nevertheless, as seen in the graph, our proposed BMNN algorithm achieved the highest energy efficiency among all algorithms, highlighting its potential to enhance system performance with a clear advantage.

## 5. Conclusions

In this research, we addressed the joint optimization problem of precoding and power allocation in massive MIMO-NOMA networks, aiming to maximize the sum rate for all devices. To tackle this challenge, we transformed the original optimization problem into an unconstrained problem for the precoding subproblem. We employed the FP approach to handle the non-convex problem, resulting in three equivalent problems and a closed expression for precoding. For the power allocation subproblem, which remains nonconvex, we utilized the MMSE-based dynamic power allocation scheme to solve it. Simulation results demonstrated that the proposed beamspace MIMO-NOMA system outperforms the baseline in terms of both spectrum and energy efficiency. In future work, we intend to extend the proposed optimization framework for precoding from beam-based optimization to user-based optimization, aiming to further improve system performance.

## Data Availability

Not applicable.

## Appendix A

When $\left\{p_{m,n}\right\}$ is held constant, the optimization problem $P_{1}$ can be transformed into the following form:

(A1) $$\begin{array}{c} P_{beam}:\underset{\left\{w_{m}\right\}}{\max}\overset{N_{RF}}{\underset{m=1}{\sum }}\overset{\left|S_{m}\right|}{\underset{n=1}{\sum }}\log_{2}\left(1+\frac{{\left\|h_{m,n}^{H}w_{m}\right\|}_{2}^{2}p_{m,n}}{\sum_{k=1}^{n-1}{\left\|h_{m,n}^{H}w_{m}\right\|}_{2}^{2}p_{m,k}+\sum_{l\neq m}^{N_{RF}}\sum_{k=1}^{\left|S_{l}\right|}{\left\|h_{m,n}^{H}w_{l}\right\|}_{2}^{2}p_{l,k}+\sigma^{2}}\right)\\ s.t.:C_{1}:\overset{N_{RF}}{\underset{m=1}{\sum }}\overset{\left|S_{m}\right|}{\underset{n=1}{\sum }}p_{m,n}{\left\|w_{m}\right\|}^{2}\leq P_{max}. \end{array}$$

Let $w_{m}=aw_{m}^{\prime }$, $\forall m\in \left\{1,2,\cdots ,N_{RF}\right\},$ $\alpha \in {\mathbb{R}}^{+}$. Then, ${\left\|h_{m,n}^{H}w_{m}\right\|}_{2}^{2}=a^{2}{\left\|h_{m,n}^{H}w_{m}^{\prime }\right\|}_{2}^{2}$, and the objective function of $P_{beam}$ can be expressed as the following expression:

(A2) $$R_{sum}=\overset{N_{RF}}{\underset{m=1}{\sum }}\overset{\left|S_{m}\right|}{\underset{n=1}{\sum }}\log_{2}\left(1+\frac{{\left\|h_{m,n}^{H}w_{m}^{\prime }\right\|}_{2}^{2}p_{m,n}}{\sum_{k=1}^{n-1}{\left\|h_{m,n}^{H}w_{m}^{\prime }\right\|}_{2}^{2}p_{m,k}+\sum_{l\neq m}^{N_{RF}}\sum_{k=1}^{\left|S_{l}\right|}{\left\|h_{m,n}^{H}w_{l}^{\prime }\right\|}_{2}^{2}p_{l,k}+\frac{\sigma^{2}}{a^{2}}}\right)$$

From (A2), it is evident that when $\left\{p_{m,n}\right\}$ and $\left\{w_{m}\right\}$ are held constant, $R_{sum}$ exhibits a monotonically increasing behavior in relation to the variable α. In other words, as $R_{sum}$ increases, resulting in an increase in ${\left\|w_{m}\right\|}^{2}$. In the case where $\left\{p_{m,n}\right\}$ is constant, as ${\left\|w_{m}\right\|}^{2}$ increases, it will eventually reach the upper bound $P_{max}$ set by constraint $C_{1}$. Therefore, to attain the maximum value of $R_{sum}$ under the constraint $C_{1}$, the equation holds, denoted as $\overset{N_{RF}}{\underset{m=1}{\sum }}\overset{\left|S_{m}\right|}{\underset{n=1}{\sum }}p_{m,n}{\left\|w_{m}\right\|}^{2}=P_{max}$.

## Appendix B

Sufficiency:

Suppose ${\bar{w}}_{m}^{o}$ is an arbitrary stationary point of unconstrained problem ${\bar{P}}_{beam}$, and let ${\tilde{w}}_{m}^{o}$ be defined as ${\tilde{w}}_{m}^{o}=\sqrt{a}{\bar{w}}_{m}^{o},\forall m,$ where $a=\frac{P_{max}}{\sum_{m=1}^{N_{RF}}\sum_{n=1}^{\left|S_{m}\right|}p_{m,n}{\left\|{\bar{w}}_{m}^{o}\right\|}^{2}}$. The sufficiency condition aims to demonstrate that ${\tilde{w}}_{m}^{o}$ is a nontrivial stationary point of the original problem ${\tilde{P}}_{beam}$.

Let ${\bar{R}}_{m,n}$ represent the achievable rate of the unconstrained optimization, which can be expressed as follows:

(A3) $${\bar{R}}_{m,n}=\log_{2}\left({\left\|h_{m,n}^{H}w_{m}\right\|}_{2}^{2}p_{m,n}+{\tilde{\xi }}_{m,n}\right)-\log_{2}{\tilde{\xi }}_{m,n}.$$

Since ${\bar{w}}_{m}^{o}$ is a stationary point of the unconstrained optimization problem ${\bar{P}}_{beam}$, we can obtain the following result:

(A4) $$\nabla_{w_{m}}\left(\overset{\left|S_{m}\right|}{\underset{n=1}{\sum }}{\bar{R}}_{m,n}\left({\bar{w}}_{m}^{o}\right)\right)+\nabla_{w_{m}}\left(\overset{N_{RF}}{\underset{x\neq m}{\sum }}\overset{\left|S_{x}\right|}{\underset{y=1}{\sum }}{\bar{R}}_{x,y}\left({\bar{w}}_{x}^{o}\right)\right)=0.$$

Observing that $\nabla_{w_{m}}{\bar{R}}_{sum}\left(tw\right)=\frac{1}{t}\nabla_{w_{m}}{\bar{R}}_{sum}\left(w\right)$ for any value t>0, Equation (A4) can be transformed into the following expression:

(A5) $$\nabla_{w_{m}}\left(\overset{\left|S_{m}\right|}{\underset{n=1}{\sum }}{\bar{R}}_{m,n}\left({\tilde{w}}_{m}^{o}\right)\right)+\nabla_{w_{m}}\left(\overset{N_{RF}}{\underset{x\neq m}{\sum }}\overset{\left|S_{x}\right|}{\underset{y=1}{\sum }}{\bar{R}}_{x,y}\left({\tilde{w}}_{x}^{o}\right)\right)=0.$$

Furthermore, we have:

(A6) $$\overset{N_{RF}}{\underset{m=1}{\sum }}\overset{\left|S_{m}\right|}{\underset{n=1}{\sum }}p_{m,n}{\left\|\sqrt{a}{\bar{w}}_{m}^{o}\right\|}^{2}=\frac{P_{max}}{\overset{N_{RF}}{\underset{m=1}{\sum }}\overset{\left|S_{m}\right|}{\underset{n=1}{\sum }}p_{m,n}{\left\|{\bar{w}}_{m}^{o}\right\|}^{2}}\overset{N_{RF}}{\underset{m=1}{\sum }}\overset{\left|S_{m}\right|}{\underset{n=1}{\sum }}p_{m,n}{\left\|{\bar{w}}_{m}^{o}\right\|}^{2}=P_{max}.$$

That is:

(A7) $$\overset{N_{RF}}{\underset{m=1}{\sum }}\overset{\left|S_{m}\right|}{\underset{n=1}{\sum }}p_{m,n}{\left\|{\tilde{w}}_{m}^{o}\right\|}^{2}=P_{max}.$$

Specifically, the gradient of the rate of the nth user in the mth beam with respect to the variable $w_{m}$ can be expressed as:

(A8) $$\nabla_{w_{m}}{\bar{R}}_{m,n}=\frac{{\left(h_{m,n}^{H}w_{m}\right)}^{*}h_{m,n}^{*}\sum_{k=1}^{n}p_{m,k}+\frac{\sigma^{2}\overset{\left|S_{m}\right|}{\underset{n=1}{\sum }}p_{m,n}{\left(w_{m}\right)}^{*}}{P_{max}}}{{\left\|h_{m,n}^{H}w_{m}\right\|}_{2}^{2}p_{m,n}+{\tilde{\xi }}_{m,n}}-\frac{{\left(h_{m,n}^{H}w_{m}\right)}^{*}h_{m,n}^{*}\sum_{k=1}^{n-1}p_{m,k}+\frac{\sigma^{2}\overset{\left|S_{m}\right|}{\underset{n=1}{\sum }}p_{m,n}{\left(w_{m}\right)}^{*}}{P_{max}}}{{\tilde{\xi }}_{m,n}}.$$

Similarly, the gradient of the rate of the nth user in the xth$\left(x\neq m\right)$ beam with respect to variable $w_{m}$ can be expressed as:

(A9) $$\nabla_{w_{m}}{\bar{R}}_{x,y}=\frac{{\left(h_{x,y}^{H}w_{m}\right)}^{*}h_{x,y}^{*}\sum_{k=1}^{\left|S_{m}\right|}p_{m,k}+\frac{\sigma^{2}\overset{\left|S_{m}\right|}{\underset{n=1}{\sum }}p_{m,n}{\left(w_{m}\right)}^{*}}{P_{max}}}{{\left\|h_{x,y}^{H}w_{x}\right\|}_{2}^{2}p_{x,y}+{\tilde{\xi }}_{x,y}}-\frac{{\left(h_{x,y}^{H}w_{m}\right)}^{*}h_{x,y}^{*}\sum_{k=1}^{\left|S_{m}\right|}p_{m,k}+\frac{\sigma^{2}\overset{\left|S_{m}\right|}{\underset{n=1}{\sum }}p_{m,n}{\left(w_{m}\right)}^{*}}{P_{max}}}{{\tilde{\xi }}_{x,y}}.$$

Since ${\tilde{w}}_{m}^{o}=\sqrt{\frac{P_{max}}{\sum_{m=1}^{N_{RF}}\sum_{n=1}^{\left|S_{m}\right|}p_{m,n}{\left\|{\bar{w}}_{m}^{o}\right\|}^{2}}}{\bar{w}}_{m}^{o},\forall m,$ (A8) and (A9) can be simplified, the expression after bringing them into (A4) can be specifically expressed as:

(A10) $$\begin{array}{c} \overset{N_{RF}}{\underset{x=1}{\sum }}\overset{\left|S_{x}\right|}{\underset{y=1}{\sum }}\frac{{\left(h_{x,y}^{H}{\tilde{w}}_{m}^{o}\right)}^{*}h_{x,y}^{*}\sum_{k=1}^{\left|S_{m}\right|}p_{m,k}}{{\left\|h_{x,y}^{H}{\tilde{w}}_{x}^{o}\right\|}_{2}^{2}\sum_{k=1}^{y}p_{x,k}+\sum_{l\neq x}^{N_{RF}}\sum_{k=1}^{\left|S_{l}\right|}{\left\|h_{x,y}^{H}{\tilde{w}}_{l}^{o}\right\|}_{2}^{2}p_{l,k}+\sigma^{2}}-\overset{N_{RF}}{\underset{x=1}{\sum }}\overset{\left|S_{x}\right|}{\underset{y=1}{\sum }}\frac{{\left(h_{x,y}^{H}{\tilde{w}}_{m}^{o}\right)}^{*}h_{x,y}^{*}\sum_{k=1}^{\left|S_{m}\right|}p_{m,k}}{{\left\|h_{x,y}^{H}{\tilde{w}}_{x}^{o}\right\|}_{2}^{2}\sum_{k=1}^{y-1}p_{x,k}+\sum_{l\neq x}^{N_{RF}}\sum_{k=1}^{\left|S_{l}\right|}{\left\|h_{x,y}^{H}{\tilde{w}}_{l}^{o}\right\|}_{2}^{2}p_{l,k}+\sigma^{2}}\\ -{\left({\tilde{w}}_{m}^{o}\right)}^{*}\overset{N_{RF}}{\underset{x=1}{\sum }}\overset{\left|S_{x}\right|}{\underset{y=1}{\sum }}\left[\frac{\frac{\sigma^{2}\overset{\left|S_{m}\right|}{\underset{n=1}{\sum }}p_{m,n}}{P_{max}}}{{\left\|h_{x,y}^{H}{\tilde{w}}_{x}^{o}\right\|}_{2}^{2}\sum_{k=1}^{y-1}p_{x,k}+\sum_{l\neq x}^{N_{RF}}\sum_{k=1}^{\left|S_{l}\right|}{\left\|h_{x,y}^{H}{\tilde{w}}_{l}^{o}\right\|}_{2}^{2}p_{l,k}+\sigma^{2}}-\frac{\frac{\sigma^{2}\overset{\left|S_{m}\right|}{\underset{n=1}{\sum }}p_{m,n}}{P_{max}}}{{\left\|h_{x,y}^{H}{\tilde{w}}_{x}^{o}\right\|}_{2}^{2}\sum_{k=1}^{y}p_{x,k}+\sum_{l\neq x}^{N_{RF}}\sum_{k=1}^{\left|S_{l}\right|}{\left\|h_{x,y}^{H}{\tilde{w}}_{l}^{o}\right\|}_{2}^{2}p_{l,k}+\sigma^{2}}\right]=0,\forall m\\ \Leftrightarrow \nabla_{w_{m}}R_{sum}\left({\tilde{w}}_{m}^{o}\right)-\tilde{\lambda }{\left({\tilde{w}}_{m}^{o}\right)}^{*}=0 \end{array}$$

where

(A11) $$\overset{N_{RF}}{\underset{x=1}{\sum }}\overset{\left|S_{x}\right|}{\underset{y=1}{\sum }}\frac{\frac{\sigma^{2}\overset{\left|S_{m}\right|}{\underset{n=1}{\sum }}p_{m,n}}{P_{max}}}{{\left\|h_{x,y}^{H}{\tilde{w}}_{x}^{o}\right\|}_{2}^{2}\sum_{k=1}^{y-1}p_{x,k}+\sum_{l\neq x}^{N_{RF}}\sum_{k=1}^{\left|S_{l}\right|}{\left\|h_{x,y}^{H}{\tilde{w}}_{l}^{o}\right\|}_{2}^{2}p_{l,k}+\sigma^{2}}-\overset{N_{RF}}{\underset{x=1}{\sum }}\overset{\left|S_{x}\right|}{\underset{y=1}{\sum }}\frac{\frac{\sigma^{2}\overset{\left|S_{m}\right|}{\underset{n=1}{\sum }}p_{m,n}}{P_{max}}}{{\left\|h_{x,y}^{H}{\tilde{w}}_{x}^{o}\right\|}_{2}^{2}\sum_{k=1}^{y}p_{x,k}+\sum_{l\neq x}^{N_{RF}}\sum_{k=1}^{\left|S_{l}\right|}{\left\|h_{x,y}^{H}{\tilde{w}}_{l}^{o}\right\|}_{2}^{2}p_{l,k}+\sigma^{2}}=\tilde{\lambda }>0.$$

This equation implies that ${\tilde{w}}_{m}^{o}$ satisfies the first-order optimality condition with respect to precoding, where $\tilde{\lambda }$ is the Lagrange multiplier. Moreover, since ${\tilde{w}}_{m}^{o}$ satisfies the power constraint and also satisfies the complementary slackness condition, it further satisfies the KKT condition of the original problem ${\tilde{P}}_{beam}$. Thus, ${\tilde{w}}_{m}^{o}$ is a nontrivial stationary point of ${\tilde{P}}_{beam}$. The sufficiency proof is finally complete.

The sufficiency of the proposition can be demonstrated by reversing the steps of sufficiency proof.
